# Establishing a pediatric acute stroke protocol: experience of a new pediatric stroke program and predictors of acute stroke

**DOI:** 10.3389/fneur.2023.1194990

**Published:** 2023-05-18

**Authors:** Kamal Phelps, Christin Silos, Susan De La Torre, Amee Moreno, Robert Lapus, Nipa Sanghani, Mary Koenig, Sean Savitz, Charles Green, Stuart Fraser

**Affiliations:** ^1^University of Texas McGovern Medical School, Houston, TX, United States; ^2^School of Biomedical Informatics, The University of Texas Health Science Center Houston, Houston, TX, United States; ^3^The UTHealth Institute for Stroke and Cerebrovascular Disease, The University of Texas Health Science Center, Houston, TX, United States; ^4^Department of Internal Medicine, University of Illinois College of Medicine, Chicago, IL, United States; ^5^Baylor University Louise Herrington School of Nursing, Dallas, TX, United States; ^6^Division of Pediatric Emergency Medicine, Department of Emergency Medicine, The University of Texas McGovern Medical School, Houston, TX, United States; ^7^Division of Child and Adolescent Neurology, Department of Pediatrics, The University of Texas McGovern Medical School, Houston, TX, United States

**Keywords:** pediatric stroke, code stroke program, acute stroke care, pediatric neurology, stroke prediction

## Abstract

**Introduction:**

Pediatric stroke is among the top 10 causes of death in pediatrics. Rapid recognition and treatment can improve outcomes in select patients, as evidenced by recent retrospective studies in pediatric thrombectomy. We established a collaborative protocol involving the vascular neurology and pediatric neurology division in our institution to rapidly diagnose and treat pediatric suspected stroke. We also prospectively collected data to attempt to identify predictors of acute stroke in pediatric patients.

**Methods:**

IRB approval was obtained to prospectively collect clinical data on pediatric code stroke activations based on timing metrics in resident-physician note templates. The protocol emphasized magnetic resonance imaging over computed tomography imaging when possible. We analyzed performance of the system with descriptive statistics. We then performed a Bayesian statistical analysis to search for predictors of pediatric stroke.

**Results:**

There were 40 pediatric code strokes over the 2.5-year study period with a median age of 10.8 years old. 12 (30%) of patients had stroke, and 28 (70%) of code stroke patients were diagnosed with a stroke mimic. Median time from code stroke activation to completion of imaging confirming or ruling out stroke was 1 h. In the Bayesian analysis, altered mental status, hemiparesis, and vasculopathy history were associated with increased odds of stroke, though credible intervals were wide due to the small sample size.

**Conclusion:**

A trainee developed and initiated pediatric acute stroke protocol quickly implemented a hospital wide change in management that led to rapid diagnosis and triage of pediatric stroke and suspected stroke. No additional personnel or resources were needed for this change, and we encourage other hospitals and emergency departments to implement similar systems. Additionally, hemiparesis and altered mental status were predictors of stroke for pediatric acute stroke activation in our Bayesian statistical analysis. However credible intervals were wide due to the small sample size. Further multicenter data collection could more definitively analyze predictors of stroke, as well as the help in the creation of diagnostic tools for clinicians in the emergency setting.

## Background

Landmark studies in thrombolysis and thrombectomy have transformed the world of acute stroke care over the past 25 years. Rapid diagnosis, thrombolysis, and thrombectomy in acute stroke are now a defining feature of the field of vascular neurology (1). Translation of these experiences to pediatric acute stroke care has been a source of significant clinical and research interest (2). Implementation of pediatric “acute stroke” protocols have been steadily increasing in use for the past decade (2–4) and recent publications have demonstrated promising results on the safety and efficacy of thrombectomy in children (5, 6). Stroke in children is split relatively evenly between acute ischemic stroke and intracranial hemorrhage, with most non-traumatic intracranial hemorrhages in children being secondary to arteriovenous malformation rupture (7). Important risk factors for acute ischemic stroke in children include focal cerebral arteriopathy, arterial dissection, complex congenital heart disease, and sickle cell disease, among many other causes (8). It is common for children with acute ischemic stroke to present with seizure, headache, and non-specific symptoms in addition to focal findings like aphasia and hemiparesis commonly described in adult stroke patients (9, 10). About 20% of acute ischemic stroke in children is posterior circulation (8, 11, 12). Childhood stroke affects ~1 in 25,000 children per year, with approximately 75% of children suffering long term neurologic morbidity (13).

Though stroke is relatively rare in children, it is an important cause of neurologic disability in pediatrics and appropriate treatment can lead to better outcomes in children, in particular with regard to thrombectomy (6, 14). Because children will have many years to live with disability from their stroke, any intervention to improve outcome can benefit the child for decades. Several pediatric centers have published clinical pathways on acute stroke (15–18), but challenges still remain in pediatric acute stroke diagnosis and treatment. Diagnosis time is often delayed, with estimates of average time from symptom onset to diagnosis as high as 24 h in recent publications (19, 20). However, implementation of a pediatric acute stroke protocol has been demonstrated to improve diagnosis time in pediatric patients that are recognized as potentially having a stroke by emergency department nurses and physicians (21).

Practical implementation of pediatric acute stroke protocols can be challenging for a variety of reasons. Because pediatric stroke is emergent and rare, there are fewer opportunities for attainment (and maintenance) of the unique skills required of hospital staff and physicians for pediatric stroke evaluation. There are also relatively few physicians with specific training in pediatric stroke, and many tertiary care children’s hospitals will not have a pediatric stroke specialist on staff (2, 22). Given the importance of rapid diagnosis and treatment in pediatric acute stroke, we created a pediatric acute stroke protocol within our children’s hospital. Children’s Memorial Hermann is a large academic tertiary care hospital with 330 beds. It did not have a dedicated pediatric stroke protocol prior to 2019. This initiative was designed and initiated by a child neurology resident and a PhD candidate with guidance from an adult stroke neurologist and child neurologist. For this project, we implemented a pediatric “code stroke” paging system, standardized order sets, and note templates, in addition to dedicated educational curriculum for pediatric stroke. In this paper, we will present data from the first 2.5 years of code strokes within our hospital system, analyze findings on presentation that may be predictors of stroke, and discuss practical challenges in implementing our code stroke system. In doing so, we hope that lessons from our experience can aid other children’s hospitals in the process of developing pediatric “code stroke” systems. Additionally, using a multivariable, Bayesian statistical analysis using assumptions based on data from previous pediatric “code stroke” papers, we analyze the clinical features of our code stroke patients for predictors of acute stroke.

## Methods

### Participants

Institutional Review Board approval was obtained to gather and analyze data on patients for which pediatric “code strokes” are paged. Pediatric patients presenting to our hospital for which a code stroke was activated were prospectively enrolled in an institutional Redcaps database. Data was analyzed for 40 children who had a code stroke called after the new protocol was implemented, from November 2019 until June 2022.

### Data collection

Data for all code stroke cases between November 2019 and June 2022 was obtained through chart review and entered into an institutional redcaps database. Note templates were used by on-call neurology residents which captured relevant clinical data.

### Clinical data

Data recorded included patient age, gender, date of admission, presenting symptoms, medical history, and final diagnosis. Patients with arterial ischemic stroke, non-traumatic intracranial hemorrhage, or cerebral venous sinus thrombosis were coded as having stroke. All other patients were coded as having a stroke mimic. In addition, stroke timing metrics including the time the patient was last seen normal (LSN), time of presentation to the hospital, time of initial imaging, time the code stroke was called, and time of diagnosis (based on time that stroke was either diagnosed or ruled out with imaging). The database contained imaging modalities utilized (computed tomography, magnetic resonance imaging) and whether the patient received sedation and if so, what type (intravenous sedatives vs. general anesthesia). Stroke etiology (cardioembolic, cryptogenic, etc.) was noted for cases diagnosed as stroke, and for confirmed strokes the use of reperfusion therapy with thrombectomy and/or alteplase was noted. Code strokes called in the emergency department were considered outpatient, and code strokes called within the medical floor, IMU, or ICU were considered inpatient.

### Acute stroke protocol

The creation of Children’s Memorial Hermann Hospital’s pediatric acute stroke protocol began as a resident-driven quality improvement project. Before the guidelines were implemented, code strokes could be requested by pediatric providers leading to arrival of an adult vascular neurology fellow that would evaluate the patient independently and decide on further action upon seeing the patient. However, no formal roles, imaging guidelines, tPA guidelines, or thrombectomy guidelines had been agreed upon for the children’s hospital. Pediatric neurologists were not part of the acute stroke decision making process, and no formal guidelines on tPA or thrombectomy in patients under 18 had been agreed upon between departments. Consequently, metrics of pediatric code strokes were not recorded prior to the quality improvement project.

The project aims were to prioritize “hyperacute” DWI centered magnetic resonance imaging over computed tomography imaging whenever possible, rapidly transfer management of non-stroke emergency consultation responsibilities back to the pediatric neurology team once stroke had been ruled out, and streamline separate provider roles for pediatric “code strokes” to ensure timely care of pediatric patients with sudden onset focal neurologic deficits.

The following were features of the code stroke protocol.

### Pediatric code stroke page

The pediatric acute stroke protocol is for code strokes in the ED and in-hospital settings. The clinical pathway diagram is available in Supplementary material. Some of the key features of the acute stroke protocol shown are:

- Two criteria should be met for a team to call a pediatric code stroke: (1) A focal neurologic deficit must be present and (2) The problem has been present for less than 24 h.
- MRI stroke imaging prioritized diffusion weighted imaging first.
- If MRI is not *immediately* available, the patient is taken for a CT and CT angiogram of the brain to rule out intracranial hemorrhage and large artery occlusion.
- Patients who are candidates for tissue plasminogen activator based on institutional guidelines receive a 0.9 mg/kg IV total dose consisting of a bolus dose (10% of total dose) over 1 min and an infusion dose (remaining 90%) over 1 h.

### Agreement on tissue plasminogen activator and thrombectomy criteria

- Patients with large artery occlusion 2 years or older who would otherwise meet criteria for adult thrombectomy based on imaging guidelines are considered for thrombectomy in discussion with the neurointerventionalist on call. This age range was agreed upon in discussion among leadership in the neurointerventional, pediatric neurology, pediatric neurosurgery, and vascular neurology divisions. The neurointerventionalists for the institution included two neurosurgeons, one neuro-interventional radiologist, and one vascular neurologist.
- Tissue Plasminogen Activator inclusion and exclusion criteria were adapted from the Thrombolysis in Pediatric Stroke trial.
- Pediatric-specific neurointerventional catheters and other equipment were made available in the adult neuroradiology suite, which is connected to the children’s hospital by an enclosed skybridge.

### Standardized documentation and order sets

Clinical note templates along with a pediatric stroke “order set” were created for use by the neurology residents and fellows in order to ensure key metrics were captured and to clarify communication between medical teams. The notes included common initial labs which could be edited by the ordering physician. Recommended workup was adapted from the 2019 AHA scientific statement on pediatric stroke. The inpatient stroke guidelines are available in Supplementary material.

### Education

Educational lectures and workshop sessions were created by child neurology residents and given to residents, fellows, and attending physicians throughout the pediatrics, emergency medicine, neurosurgery, and neurology departments. Importantly, pediatric stroke was added to the foundational “boot-camp” series of lectures on neurologic emergencies that were given to neurology residents, child neurology residents, and vascular neurology fellows. Pediatric stroke was added to pediatrics and emergency medicine core lecture series for residents. The lectures emphasized typical signs and symptoms of pediatric stroke and how they differ from adults, differential diagnosis of pediatric acute focal neurologic deficits as based on prior published data, the importance of obtaining MRI imaging over CT if feasible, and how to activate the pediatric code stroke alert.

### Data analysis

Confirmatory analysis of possible clinical stroke predictors, used a Bayesian approach with horseshoe prior distributions for regularization. Regularization will result in more conservative estimates, increasing the likelihood of replication in future samples (23). A medical history of vasculopathy or prior stroke, as well as dysarthria, altered mental status, or hemiparesis on exam had previously been noted to be significant predictors of stroke in prior pediatric code stroke related studies (15, 16, 24). These factors were used in the prior distribution used for the statistical analysis.

After the confirmatory analysis, an exploratory analysis was completed to find unexpected predictors of stroke for code stroke patients.

## Results

There were 40 pediatric code strokes from November 2019 until June 2022 including 28 outpatient and 12 inpatient cases. Of these patients, 63.4% were male, with a median age of 10.8 years old. Table 1 shows the presenting symptoms found in the 40 patients.

**Table 1:** Baseline demographics and timing metrics of code stroke patients.

	All	Stroke	Non-stroke
N (%)	40	12 (30%)	28 (70%)
Age (years)	10.8	8.6	11.7
Male sex	26 (63.4%)	9 (75%)	17 (58.6%)
Median time to first medical contact (hours)	3.5	7	3.3
Median time from presentation to first image (hours)	1.33	1	1.65
Median time from code stroke to first image (hours)	0.8	0.7	1
First imaging type (MRI)	28 (70%)	8 (66.7%)	20 (71.4%)
Anesthesia/Sedation (yes)	8 (20%)	4 (25%)	4 (14.3%)
*Presenting symptoms*			
Hemiparesis/Hemiplegia	24 (60%)	8 (66.7%)	16 (57.1%)
Hemisensory loss	11 (27.5%)	2 (16.7)	9 (32.1%)
Seizure	8 (20%)	2 (16.7%)	6 (21.4%)
Aphasia	9 (22.5%)	3 (25%)	6 (21.4%)
Dysarthria	6 (15%)	1 (8.3%)	5 (17.9%)
Altered mental status	12 (30%)	6 (50%)	6 (21.4%)
Facial droop	14 (35%)	4 (33.3%)	10 (35.7%)
Ataxia	3 (7.5%)	1 (8.3%)	2 (7.1%)
*Medical history*			
Congenital heart disease	4 (10%)	3 (25%)	1 (3.6%)
Malignancy	4 (10%)	2 (16.7%)	2 (7.1%)
Sickle cell	3 (7.5%)	1 (8.3%)	2 (7.1%)
Rheumatological	1 (2.5%)	0	1 (3.6%)
Recent trauma	3 (7.5%)	1 (8.3%)	2 (7.1%)
Prior stroke	2 (5%)	0	2 (7.1%)
Vasculopathy	3 (7.5%)	2 (16.7%)	1 (3.6%)

**Figure 1:**
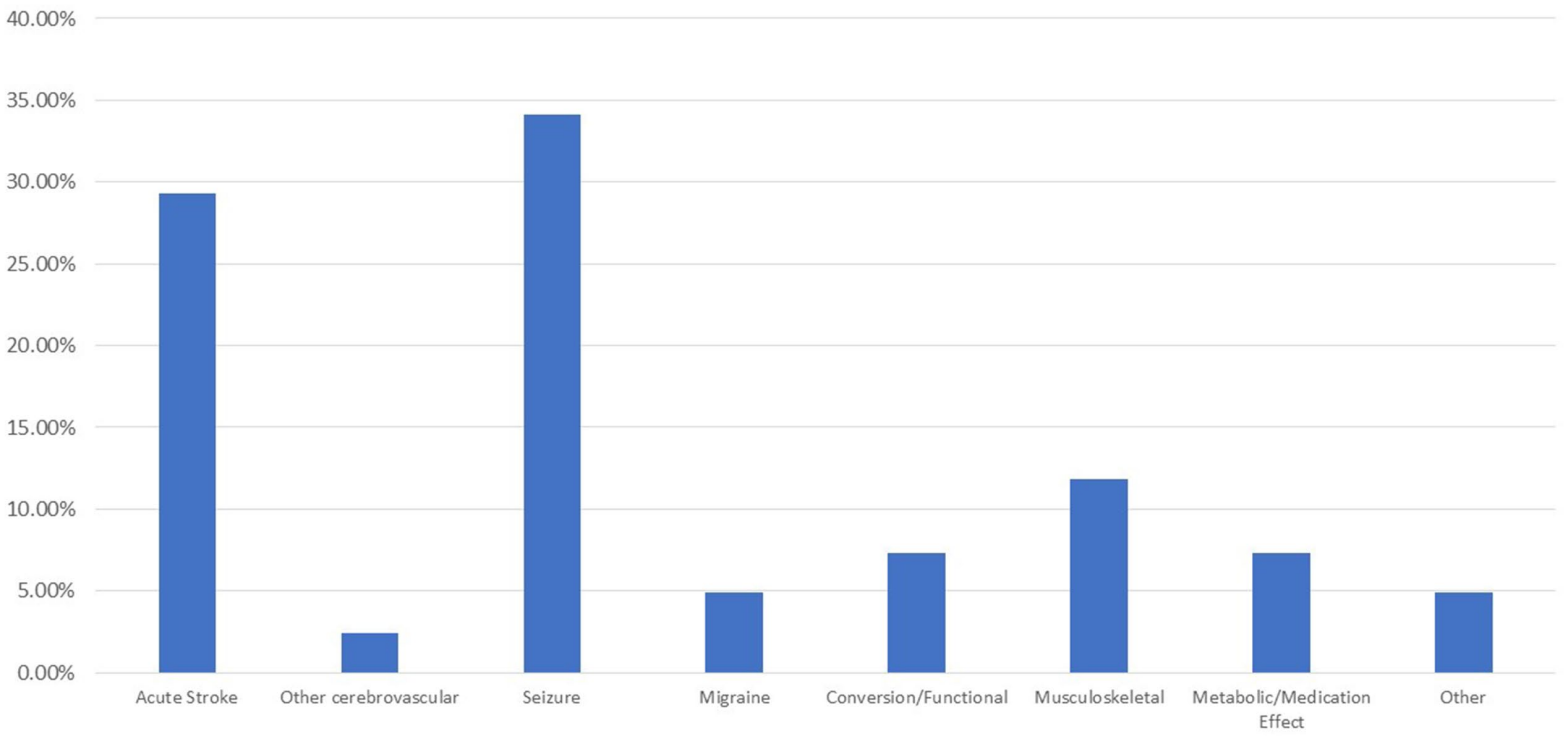
Final diagnosis by stroke type.

Final diagnoses are demonstrated in Figure 1. Ten of the code stroke patients had acute ischemic stroke, one had cerebral venous sinus thrombosis, and one patient suffered a spontaneous intracranial hemorrhage from arteriovenous malformation rupture. No patients required decompressive hemicraniectomy for malignant infarction during the study period. Of the ischemic stroke patients within the study period, two met criteria for acute reperfusion therapy at presentation. One patient received tPA and thrombectomy with complete recovery. However, the other patient did not have a code stroke called until the morning when the patient was seen by the attending cardiologist. At the time imaging was obtained the infarct was complete, and the patient did not receive a thrombectomy or tissue plasminogen activator. Of the non-cerebrovascular final diagnosis patients, 14 (34.1%) ultimately were diagnosed with a seizure, 2 (5%) with migraine, 3 (7.5%) with a functional neurologic disorder, 4 (8.9%) with musculoskeletal disorder, 3 (7.3%) with a metabolic or medication effect, and 2 (4.9%) had another disorder not applicable to the other categories. One patient presented with reversible cerebral vasoconstriction syndrome and was coded as “other cerebrovascular.”

Timing from onset of symptoms to imaging and treatment is crucial to decrease morbidity from stroke. In our data set, the median time from reported last known well until first medical contact was 3.5 h for all patients, and was longer for stroke patients at 7 h. There were several outlier patients, with delays as long as 40 h, which is why a median was calculated rather than mean.

### Analysis of stroke vs. non-stroke cases

We compared the clinical characteristics between the stroke (*n* = 12) and non-stroke (*n* = 28) cases. Median Pediatric National Institute of Health Stroke Scale (PedNIHSS) was available for 5 stroke patients and 4 for non-stroke patients. The median stroke patient age (8.6 years) was notably less than the non-stroke patients (11.7 years) and both groups showed a greater ratio of males (75% stroke, 58.6% non-stroke). There was a notable increase in the time delay between LSN and presentation for the stroke patients (7 h) compared to their non-stroke counterparts (3.3 h). However, other time delays were similar in the two groups, including presentation to imaging, code stroke to imaging, and presentation to code stroke. Additionally, imaging utilization was comparable in both groups, with MRI imaging being the most common modality (66.7% stroke, 71.4% non-stroke). In stroke patients, the most common presenting symptoms were hemiparesis (66.7%), altered mental status (50%), and facial weakness (33.3%). In contrast, non-stroke patients most commonly presented with hemiparesis (57.1%), speech changes such as dysarthria or aphasia (39.3%), changes in sensation such as loss or numbness (32.1%), and facial weakness (35.7%).

### Use of anesthesia and code stroke timing delays

Analysis of sedation used for imaging revealed 80% of all patients received none, 10% received general anesthesia, and 10% received IV sedation. Table 2 contains the timing delays encountered between code stroke to imaging and code stroke to diagnosis, broken down by the type of anesthesia used for all 40 code stroke cases. The median timing from code stroke to imaging for patients who received general anesthesia was 1.7 h, which is longer than those who received IV sedation (1.65 h) and no anesthesia (0.8 h). The median timing from code stroke to diagnosis was also notably longer for patients who underwent general anesthesia (9.9 h) compared to IV sedation (1.65 h) and no anesthesia (1 h).

**Table 2:** Code stroke delays when using anesthesia.

	None	General anesthesia	IV sedation
Median timing from code stroke to first imaging (hours)	0.8	1.7	1.65
Median timing from code stroke to confirmatory diagnostic imaging (hours)	1	9.9	1.65

### Bayesian analysis for predictors of stroke

The confirmatory Bayesian analysis (Table 3) found associations for hemiparesis, altered mental status, and vasculopathy with increased odds of stroke. Dysarthria on presentation and a prior history of stroke were associated with decreased odds of stroke. However, the 95% credible value for all of these values crossed null. In the exploratory analysis, the 95% credible intervals were similarly large, and all values crossed null. The area under the curve (AUC) for the confirmatory analysis was 0.70. The AUC for the exploratory analysis was 0.86. The odds ratios and 95% credible intervals for the exploratory analysis are presented in Table 4.

**Table 3:** Confirmatory Bayesian analysis of predictors of stroke.

	Estimate (odds ratio)	Estimated error	Q2.5	Q97.5
Intercept	0.28	1.88	0.07	0.8
Prior stroke	0.58	3.46	0.02	3.11
Vasculopathy history	2.16	3.03	0.53	34.25
Dysarthria	0.7	2.15	0.1	2.29
Altered mental status	2.05	2.1	0.76	10.81
Hemiparesis	1.28	1.73	0.51	4.93

**Table 4:** Exploratory Bayesian analysis of predictors of stroke.

	Estimate (odds ratio)	Estimated error	Q2.5	Q97.5
Intercept	0.19	4.03	0.01	2.48
Age	0.98	1.08	0.81	1.13
LSN to hospital presentation (hours)	1.04	1.04	0.99	1.13
Presentation to imaging time (hours)	1.09	1.11	0.95	1.39
IV sedation	0.84	2.04	0.11	2.24
No sedation	0.8	1.94	0.1	1.66
Hemiparesis	1.05	1.48	0.5	2.88
Hemisensory loss	1.03	1.53	0.45	2.94
Seizure	0.79	2.13	0.08	1.8
Aphasia	1.29	1.92	0.64	9.88
Dysarthria	0.97	1.52	0.33	2.27
Altered mental status	2.16	2.98	0.83	38.18
Facial droop	0.99	1.48	0.4	2.46

## Discussion

We studied our institution’s implementation of a pediatric acute stroke protocol which consisted of a pediatric code stroke paging system, standardized order sets, clinical note templates, and educational lectures for faculty, residents, fellows, and staff. Our study aims to report data on important code stroke timing metrics to establish an operating baseline, acknowledge successes, and identify areas for improvement. Regarding ischemic stroke risk factors, 75% of all stroke patients in our study were male which agrees with the male predominance noted in other studies (25). Also, the risk factors and etiologies documented for 4 of the ischemic stroke cases in our study were consistent with those most often identified in the literature. Specifically, these risk factors and etiologies were congenital heart disease, head and neck infection, and sickle cell disease (8). Notably, code strokes were rarely called for intracranial hemorrhages, despite that nearly half of childhood stroke is hemorrhagic (7).

The code stroke metrics analysis shows that inpatients had longer delays on average for imaging and evaluation. We suspect the higher functionality and better health typically displayed by children outside the hospital could partly explain the shorter time between LSN and presentation. The higher level of activity and better health at baseline may aid in timely recognition when a deficit presents. Also, it may be more challenging for hospitalized children to express changes in their neurologic functionality or struggle to distinguish it from their concurrent medical condition(s). The prolonged in-patient delays are more puzzling and indicate that it takes longer for hospitalized patients to have a code stroke called, receive imaging, and receive a diagnosis. Possible explanations worth considering include poorer baseline neurologic status in hospitalized patients, particularly ICU patients, the lengthy process of completing urgent inpatient imaging, and the staffing familiarity differences in the pediatric floor and ICU as compared to the emergency department.

An essential feature of enacting our code stroke protocol is education to nursing staff, residents and faculty in the divisions of emergency medicine, vascular neurology, child neurology, pediatric hospitalist medicine, and pediatric intensive care. In these lectures and workshops, we discussed the importance of completing MRI imaging first when possible. MRI imaging is highly sensitive for detecting pediatric strokes and is considered first-line in the absence of contraindications (3, 20). This can be confusing for providers whose medical education and training typically emphasizes performing CT imaging first in suspected adult stroke cases. Unfortunately, the workup and diagnosis of pediatric stroke are not as widely taught. Our study showed that MRI/MRA imaging was obtained first in 70% of the code strokes. Pre-protocol, all imaging was CT/CTA at the discretion of the ordering stroke neurologist. We believe these results indicate that most of our providers are first considering MRI imaging when a child presents with a suspected stroke. CT imaging was preferred in certain patients for reasons including the presence of a pacemaker or bullet, medical instability, or inability to remain still for MRI. In 3 cases in the early period of the protocol implementation, CT imaging was ordered first despite no contraindications. To ensure the best possible care for new acute stroke patients, pediatric stroke protocols are now considered part of our institution’s resident “boot camp” in multiple departments, and refreshers courses are regularly given to faculty. As pediatric stroke is infrequent but emergent, we feel repeated and relatively frequent staff education is an important feature of any new or established pediatric stroke program.

The use of initial MRI imaging when feasible may reduce unnecessary dual imaging, prevent unnecessary radiation exposure, reduce medical costs for the patient, and lead to a timelier stroke diagnosis. However, flexibility is an important part of pediatric stroke evaluation. A review of the house staff consult notes revealed specific clinical situations that made MRI unattainable or clinically unwise to obtain first. The stroke team determined that one patient’s facial droop was due to a cranial nerve VII palsy from prior trauma, and MRI was subsequently deferred. Another patient had recent surgery for moyamoya disease; therefore, neurosurgery strongly recommended CT imaging to rapidly exclude a post-surgical complication. Lastly, in a patient with a ventriculoperitoneal shunt, CT imaging was best for quickly ruling out dysfunction of the shunt in an agitated patient. These cases demonstrate that physicians should follow the acute stroke response guidelines, but many clinical scenarios warrant a more tailored approach.

The most common risk factor identified in the stroke cohort was congenital heart disease (25%), one of the most prevalent risk factors documented in the literature. Workup for the two patients revealed a cardioembolic etiology related to their underlying conditions. One patient in the cohort had sickle cell disease identified as a risk factor, but many patients (50%) had no identifiable risk factors at presentation. Providers should be on the lookout for risk factors of pediatric stroke and have a higher level of suspicion when those patients present with atypical neurologic symptoms or are unable to express their symptoms. For example, our stroke team responded to a case where a 5-year-old patient with congenital heart disease was on the pediatric floor when she presented with dysphagia and facial droop. The responding team did not initially suspect a stroke, which resulted in delayed activation of the stroke response protocol (16.7 h). Ultimately, the patient was diagnosed with an ischemic infarct of the left middle cerebral artery. This case encourages us to continue hosting educational meetings for staff members and providers likely to be the responding physician when a patient presents with a possible stroke.

In our Bayesian analysis looking at predictors of stroke from our code stroke data, we found that our data generally agreed with the three prior studies on clinical predictors of acute stroke in pediatric patients, including hemiparesis, altered mental status, and a history of vasculopathy (15, 16, 24). The AUC for the exploratory model was 0.86, indicating excellent prediction of stroke. However, the 95% credible intervals for most variables were large owing to the small sample size. Consequently, the model should be interpreted with caution, and would benefit from larger sample size in the future. Notably, the model is Bayesian permitting updated parameter estimates with the acquisition of more data. We feel this indicates the need for more research of clinical predictors of acute stroke in pediatric patients. Multicenter prospective studies, either through the International Pediatric Stroke Study or data use agreements between institutions, represent promising avenues of further research in developing tools to aid clinicians in pediatric acute stroke.

## Conclusion

In closing, this trainee-developed and initiated pediatric acute stroke protocol demonstrated a hospital wide change in stroke management that was maintained over the 31 months of data collection. This is an encouraging finding given that the protocol utilized only staff and equipment already available within the hospital, leading to improved patient care at minimal cost to the academic center. Pediatric hospitals across the country can benefit from literature review and discussions within and with external experts to improve their acute stroke care. The efficacy of stroke diagnosis following this protocol’s development allowed for a streamlined diagnostic decision-making process as the urgent magnetic resonance imaging allowed the stroke team to quickly sign off for those cases that ended up being stroke mimics.

Additionally, despite the notion that an MRI would take longer to complete due to the need of anesthesia, the use of a fast sequence MRI removed the need for sedation in the majority of cases, as only 7 of the 40 patients required sedation for imaging. Overall implementation of a pediatric acute stroke protocol can be made quickly and smoothly with positive patient outcomes. As shown by the case below, stroke can be a devastating, life changing event in childhood. However, if hospital staff can prepare for strokes and learn what the common symptoms are utilizing the best possible guidelines, outcomes can greatly improve without requiring significant additional resources.

Finally, altered mental status, hemiparesis, and a history of vasculopathy were predictors of stroke in our statistical analysis. Future, collaborative pediatric stroke studies could use this data to develop more accurate predictors of intervenable pediatric stroke. This could be used to develop clinical tools to aid emergency providers in the future.

### Brief case summary

The below example case summary highlights how implementing an acute stroke protocol can allow for the timely diagnosis and treatment of acute ischemic stroke. The patient was a 6-year-old male with a history of tricuspid atresia, absent pulmonary valve, hypoplastic right ventricle, atrial septal defect status-post fenestrated Fontan procedure, who had a pacemaker in place and was prophylactically taking 81 mg Aspirin daily. He presented to the emergency department (ED) 30 min after the sudden onset of confusion and an episode of urinary incontinence. His father endorsed similar intermittent episodes of confusion over the past week which would quickly resolve without any intervention. He had no known history of stroke and no functional deficits at baseline according to his mother.

**Figure 2:**
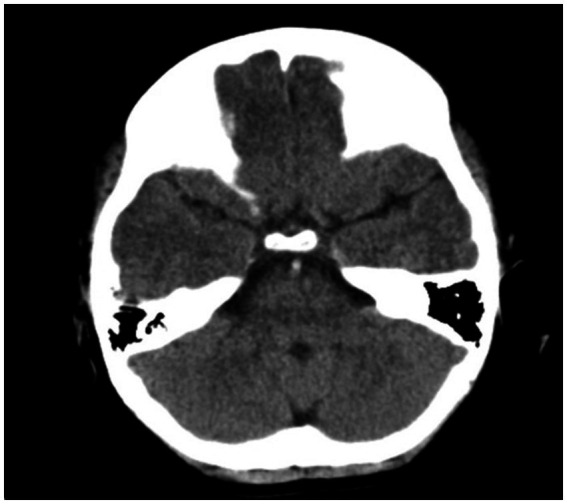
Axial CT brain revealing hyperdense basilar artery sign.

**Figure 3:**
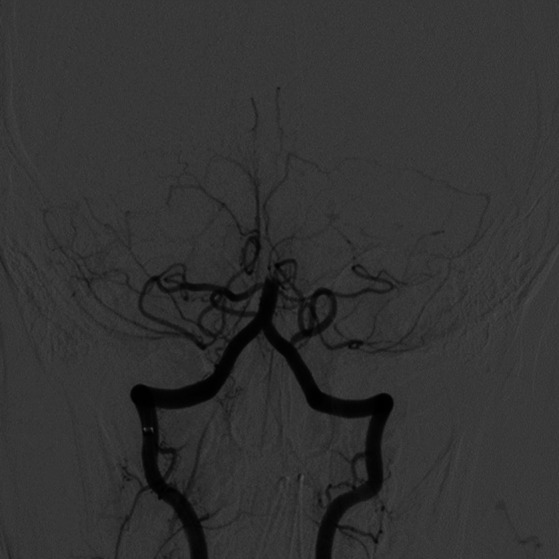
Angiogram of the vertebrobasilar system revealing basilar occlusion.

**Figure 4:**
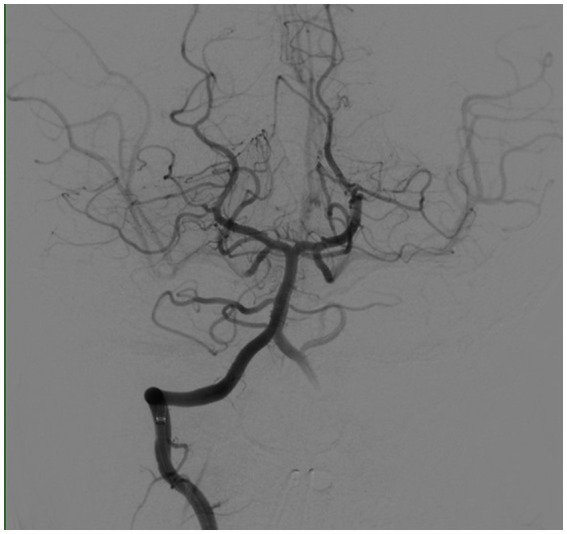
Angiogram of the vertebrobasilar system post-thrombectomy.

The patient was given Keppra and watched in the ER as he was presumed to have had a seizure, but his mental status worsened after 2 h. A pediatric code stroke was activated. Neurologic examination revealed a somnolent mental status, aphasia, left facial droop, and left upper and lower extremity weakness. CT brain without contrast revealed a hyperdense basilar artery sign (Figure 2), as well as a subacute infarct in the right posterior cerebral artery territory, along with a chronic infarct of the left middle cerebral artery territory. The stroke consult service calculated a PedNIHSS of 12. MRI could not be performed due to the presence of a pacemaker, and CTA head/neck confirmed a mid and distal basilar artery occlusive clot with poor opacification of the superior cerebellar artery origins and left anterior inferior cerebellar artery. The decision to give tPA and proceed to thrombectomy was made immediately after the CTA was completed. The patient’s mentation started to decline, he was administered 0.9 mg/kg tPA 63 min after the code stroke was called, and intubated out of concern for airway protection. Neurointerventional radiology successfully performed endovascular thrombectomy with TICI 3 recanalization 118 min after the code stroke was called (Figures 3, 4).

MRI brain performed with cardiac electrophysiology revealed small areas of diffusion restriction in the pons, however the patient was extubated the following day and found to have a PedNIHSS of 1 for dysarthria. The patient was discharged home on long term Lovenox 10 days later with no residual deficits, as his dysarthria had resolved. He continues to follow in pediatric stroke clinic 2 years after his stroke. His neurologic exam remains normal, and he has had no repeated strokes.

## Data availability statement

The original contributions presented in the study are included in the article/Supplementary material, further inquiries can be directed to the corresponding author.

## Ethics statement

The studies involving human participants were reviewed and approved by UT Health Institutional Review Board. Written informed consent from the participants’ legal guardian/next of kin was not required to participate in this study in accordance with the national legislation and the institutional requirements.

## Author contributions

KP entered input data into the database and was the primary writer of the manuscript. CS performed data analysis and wrote part of the methods section. SD entered data into redcaps and wrote part of the manuscript introduction and results. AM entered data and also edited the manuscript. RL and NS helped design the clinical protocol and edited the manuscript. MK helped design and implement the clinical protocol. SS helped design and implement the clinical protocol and edited the manuscript for content. CG supervised and edited the statistical analysis used for the final results. SF designed, implemented, and oversaw the new code stroke project, entered data into the redcaps database, and edited the final manuscript. All authors contributed to the article and approved the submitted version.

## Conflict of interest

The authors declare that the research was conducted in the absence of any commercial or financial relationships that could be construed as a potential conflict of interest.

## Publisher’s note

All claims expressed in this article are solely those of the authors and do not necessarily represent those of their affiliated organizations, or those of the publisher, the editors and the reviewers. Any product that may be evaluated in this article, or claim that may be made by its manufacturer, is not guaranteed or endorsed by the publisher.
