# Supplementary material for: Establishing a pediatric acute stroke protocol: experience of a new pediatric stroke program and predictors of acute stroke
Source: Front Neurol. 2023 May 18;14:1194990. doi: 10.3389/fneur.2023.1194990 (PMC10232855; doi:10.3389/fneur.2023.1194990)
Supplement: Supplementary file 1 [file Data_Sheet_1.PDF]

Supplementary Material

Pediatric Acute Stroke Protocol

Page 1

Pediatric Acute Stroke Protocol

Child 0 -17 years old with concern for acute stroke

Note:
Patients with known MELAS have a separate protocol for stroke-like episodes

For patients with MELAS presenting with focal neurologic deficits, **page pediatric neurology and see MELAS protocol**

Patient meets **BOTH acute stroke criteria** :
1. There is a focal neurologic deficit
   a. Unilateral weakness or sensory change
   b. Painless vision loss
   c. Dysarthria or aphasia
   d. Nystagmus or ataxia
2. The problem has been present for 24 hours or less

No

Page Pediatric Neurology

The Pediatric Neurology team will see the patient and consult Dr. Stuart Fraser (or, if he is unavailable, the adult stroke team) if indicated

Yes

ED or Responsible Service:
1. Call the page operator and ask to page **Pedi Code Stroke** , the patient's location, and call back number
2. Start IV x2 (one dedicated IV for tPA)
3. Draw Stat CBC, BMP, PT/PTT/INR, Glucose
4. Initiate Neuroprotective care:
   a. Normotension (between 50 th- 15% above the 95 th %ile for age)
   b. Normoglycemia, Normothermia, Normovolemia
   c. Seizure Control. Stat AED if suspected Seizure
5. Bedside nurse completes MRI screening form STAT
6. Order and prepare patient for transport for **MRI Brain Stroke Limited + MRA Brain wo contrast +/ - MRA neck wo contrast**

Note:
Consider activating rapid response if patient is on wards to facilitate IV placement and rapid transport to imaging

Please Note:
Patients found to have ischemic stroke, CVST, or intracranial hemorrhage should generally have pediatric neurology, pediatric neurosurgery, and the pediatric hematology services consulted.

Go to Page 2

Page 2

Child neurology fellow and/or stroke fellow + overnight neurology resident confirms suspected stroke
NIHSS performed
Neurology resident/fellow or ED/wards staff calls MR scanner for emergent limited MRI
***If MRI is not *IMMEDIATELY* available or obtainable, proceed to CT/CTA**

Note:
The use of sedation/intubation for neuroimaging is up to the discretion of the primary pediatrics/ER/ICU team attending.

In general, emergent intubation for MRIs for code strokes is *NOT* recommended.

MRI Stroke Limited + MRA Performed +/- MRV +/- MR perfusion

Patient transport to CT for STAT:
- CT Brain
- CTA Brain and Neck
- *CONSIDER* CTP in teenagers

MRI stroke limited sequences should be obtained in the following order: DWI, ADC, GRE, T2 Flair, followed by MRA Brain.
Can obtain MRA Neck if considering endovascular therapy and/or MRI perfusion if considering endovascular therapy.

MRI Brain with DWI changes consistent with acute ischemic stroke
Negative for hemorrhage
MRA consistent with vascular occlusion

CT Brain normal **AND** CTA/CTP consistent with acute vascular occlusion

Contact pediatric neurosurgery if blood seen on imaging

ENDOVASCULAR THERAPY:
Thrombectomy can be pursued in select cases of pediatric patients **greater than 2 years of age** with large artery occlusion and favorable imaging within 24 hours of symptom onset

The stroke fellow, pediatric stroke attending (Dr. Fraser) or stroke attending can discuss these cases with the neuro -endovascular fellow on call as indicated

Stroke fellow/child neurology fellow, or neurology resident confirms image read with radiology attending over phone
Stroke fellow/child neurology fellow, or neurology resident confirms intention to treat with tPA with stroke attending **and** pediatric neurology attending

If requested by the stroke fellow, stroke attending, or pedi neuro attending at *any point, please don't hesitate* to call the pediatric vascular neurology attending, Dr. Fraser, who will answer any questions if available.

Go to Page 3

Page 3

tPA CONTRAINDICATIONS:

History:

- >4.5 hours from last seen well
- Stroke, major head trauma, or intracranial surgery in the last 3 months
- History of known AVM or aneurysm
- History of prior ICH
- Major surgery or biopsy within 10 days
- GI or GU bleeding within 21 days
- Patients with known malignancy or within one month of completing treatment
- Patient with underlying major bleeding disorder
  - von Willebrand, mild platelet dysfunction does not exclude
- Previously diagnosed primary angiitis of CNS or secondary arteritis
- Patient would decline blood transfusion if indicated
- Clinical presentation consistent with MI or pericarditis requiring eval by cardiology
- Arterial puncture at noncompressible site or lumbar puncture within the last 7 days. Cardiac cath via a compressible artery within 7 days are NOT excluded

Etiology:

- Stroke due to **bacterial embolism, sickle cell, meningitis, bone, air, or fat embolus, or moyamoya disease**

Exam:

- Persistent SBP >15 th% above the 95 th percentile
- Mild deficit (PedNIHSS <6)
- PedNIHSS >25, suggesting large infarct

Imaging

- CT with hypodensity/sulcal effacement
- Infarct size estimate to be greater than 1/3 rd of MCA territory
- Intracranial arterial dissection

Labs:

- Glucose <50 or >400
- Bleeding diathesis including platelets <100,000, PT >15 seconds, PTT > upper limit of normal range

tPA Treatment Protocol
Children's Memorial Hermann Hospital

tPA Candidate:
<4.5 hours from onset
Age ≥ 2 years
Persistent focal deficit
No contraindications
BOTH Proven vascular occlusion **AND**
either proven infarct on MRI
OR high suspicion of ischemic stroke based on neurologic evaluation

↓

Dose:
Total dose: 0.9mg/kg IV (max dose= 90mg)
Bolus dose: 10% of total dose, IV over 1 minute. Bolus given by neurology fellow or resident
Infusion Dose: Remaining 90% IV infusion over 1 hour
11AM-11PM: ED pharmacy prepares tPA
Off hours – bedside nurse prepares tPA

MD, nurse, and charge nurse double check dose

↓

Maintain cardiorespiratory and BP monitoring during infusion
Maintain BP between 50 th %ile and 15% above the 95 th %ile
(see page 5 for BP control protocol)
Dedicated IV required for tPA administration

↓

Patient must be monitored in PICU setting for **at least** first 24 hours after infusion
Exception:

- Patients aged ≥15 may be monitored in the adult stroke unit or neuro ICU based on discussion with pediatric neurology attending, stroke attending, the patient and the patient's family.

Page 4

BP Parameters for tPA infusion
Children's Memorial Hermann Hospital

Systolic BP should be maintained between 50th %ile for age and 15% above the 95 th %ile for age

Treat to lower BP if >15% above 95 th %ile for age

↓

Labetalol 0.2mg/kg IV push over 2-3 minutes, repeat q15 minutes
Can consider nicardipine drip, 1mcg/kg/min, titrate to desired BP

Systolic Blood Pressure Parameters -Female

Age	50%	95%	>15% above 95%
2-5 years	90	111	128
6-10 years	96	121	139
11-17 years	105	131	151

Systolic Blood Pressure Parameters- Male

Age	50%	95%	>15% above 95%
2-5 years	90	112	129
6-10 years	96	121	139
11-17 years	110	140	161
